# Brazilian version of the Pediatric Functional Status Scale: translation and cross-cultural adaptation

**DOI:** 10.5935/0103-507X.20180043

**Published:** 2018

**Authors:** Vivianne Camila de Souza Bastos, Arthur Augusto Lima Carneiro, Marina dos Santos Ramos Barbosa, Lívia Barboza de Andrade

**Affiliations:** 1 Instituto de Medicina Integral Prof. Fernando Figueira - Recife (PE), Brasil.; 2 Hospital Esperança Recife, Rede D'Or São Luiz - Recife (PE), Brasil.

**Keywords:** Semantics, Intensive therapy, Translations, Validation studies, Surveys and questionnaires, Child, Intensive care units

## Abstract

**Objective:**

To translate and cross-culturally adapt the Functional Status Scale for hospitalized children into Brazilian Portuguese.

**Methods:**

A methodological study of the translation and cross-cultural adaptation of the Functional Status Scale was conducted, according to the stages of translation, synthesis of translations, back-translation, synthesis of back-translations, expert committee analysis and pre-test with a sample of the target population. During the evaluation by the committee of experts, semantic, content and item analyses were performed.

**Results:**

The semantic, idiomatic, cultural and conceptual equivalences between the translated version and the original version were obtained, resulting in the Brazilian version of the Functional Status Scale. After the analysis by the expert committee, there were no problems regarding the cultural or conceptual equivalences because the items were pertinent to the Brazilian culture and few terms were modified. In the pre-test stage, the scale was applied by two evaluators to a sample of 25 children. Clarity and ease in answering the scale items were observed. Good inter-observer reliability was obtained, with an intraclass correlation coefficient of 0.85 (0.59 - 0.95).

**Conclusions:**

The Functional Status Scale for pediatric use was translated and culturally adapted into Portuguese spoken in Brazil. The translated items were pertinent to the Brazilian culture and evaluated the dimensions proposed by the original instrument. Validation studies of this instrument are suggested to make it feasible for use in different regions of Brazil.

## INTRODUCTION

Due to advances in intensive care in pediatric intensive care units (ICUs), there has been a reduction in mortality,^(1)^ but due to immobility, longer mechanical ventilation time and the use of drugs such as corticosteroids, neuromuscular blockers and sedatives, the risk of physical and/or neurocognitive sequelae has increased.^(2,3)^ In the short term, children in ICUs may develop muscle weakness^(4,5)^ and *delirium*^(6)^ and, in the long term, decreased functionality and difficulties in activities of daily living, school performance and social interaction.^(7)^

These morbidities may be subsequent to the underlying disease or associated with care administered in pediatric ICUs.^(8)^ For this reason, interest in the functional outcomes of this population is increasing.^(9,10)^ Instruments that evaluate the functionality of children after discharge from pediatric ICUs have been used^(11-13)^ and can identify changes early, which favors rehabilitation strategies for the dysfunctions acquired during hospitalization.^(14,15)^

There are instruments used to evaluate functional outcomes during hospitalization, but many of these instruments are not yet available in Brazil because they are not validated for the Portuguese language. Examples include the Pediatric Overall Performance Category, Pediatric Cerebral Performance Category^(16)^ and Functional Status Scale (FSS).^(17)^ A functionality scale that has already been translated and is used in Brazil is the Pediatric Evaluation of Disability Inventory (PEDI), but because it is very extensive and complex, it is infrequently used in the hospital setting.^(18)^

Among the instruments not validated for the Portuguese language, the FSS for use in children is conceptually based on scales of activities of daily living and adaptive behavior.^(17)^ The FSS has been widely used,^(19,20)^ and its objective is to evaluate the functional outcomes of hospitalized pediatric patients. It is suitable for a broad age group, easy to perform, multidisciplinary, objective and able to evaluate various clinical outcomes.^(21)^

Because of its characteristics, the FSS is a promising tool for evaluating functionality in children. However, for an instrument to be used in clinical practice, it is fundamental that it be translated and validated for the Brazilian population. The process of translation and cross-cultural adaptation is not limited to the simple translation of the original because the social, cultural and linguistic characteristics may not be well understood when translated literally into Portuguese spoken in Brazil.^(22-24)^

Given the importance of better understanding the functional performance of children after discharge from pediatric ICUs and the need to use instruments adapted to the Portuguese language, the objective of this study was to translate and cross-culturally adapt the FSS for hospitalized children into Brazilian Portuguese.

## METHODS

This was a methodological study involving the translation and cross-cultural adaptation of the FSS into the Portuguese language spoken in Brazil. Prior consent was requested and obtained from the original author of the FSS for the development of this instrument. The present study was carried out at the *Instituto de Medicina Integral Prof. Fernando Figueira* (IMIP), located in Recife, Pernambuco, and approved by the institutional Research Ethics Committee under number 2,062,654.

### Description of the Functional Status Scale

The FSS is a freely accessible scale (available at https://www.ncbi.nlm.nih.gov/pmc/articles/PMC3191069/) consisting of six domains (mental status, sensory functioning, communication, motor functioning, feeding and respiratory status). Each domain is scored on a scale from 1 point (normal) to 5 points (very severe dysfunction). The total score ranges from 6 - 30 points, and lower scores indicate better functionality. The global FSS score is categorized as follows: 6 - 7, adequate; 8 - 9, mild dysfunction; 10 - 15, moderate dysfunction; 16 - 21, severe dysfunction; and more than 21 points, very severe dysfunction.^(17)^ There are a total of 30 items. In addition to the 30 scale items, it was necessary to analyze all the terms and categories used, thus totaling 41 items and terms for analysis.

### Translation and cross-cultural adaptation

**Figure 1:**
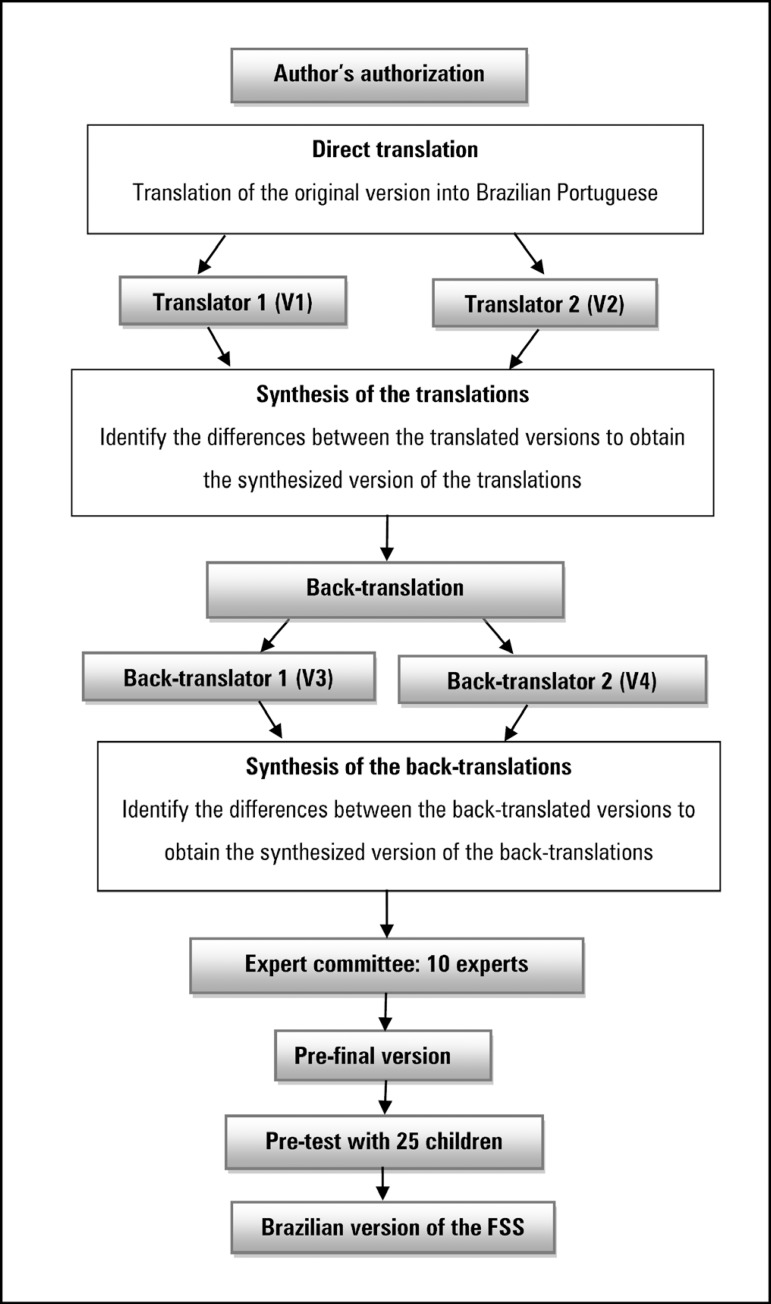
Flowchart of the translation and cross-cultural adaptation process of the Functional Status Scale. FSS - Functional Status Scale.

The process of the translation and cross-cultural adaptation of the scale into Brazilian Portuguese was based on the stages proposed by Reichenheime and Moraes,^(23)^ as shown in the flowchart in figure 1.

The translation was performed by two independent bilingual translators, whose native language was Brazilian Portuguese, generating two translated versions of the English language instrument into Brazilian Portuguese (V1 and V2). After the translations, the authors compared the two versions to identify discrepancies, and a single version, a synthesis of the Portuguese translations (STV), was elaborated.

For the back-translation, the STV was sent to two translators, whose native language was English, generating the retranslated versions (V3 and V4), which, after being compared by the authors, resulted in the synthesized version of the back-translations (SBTV).

After this stage, the six versions (V1, V2, STV, V3, V4 and SBTV) were compared to the original scale by a panel of experts composed of ten health professionals specializing in pediatrics and intensive care. They evaluated each item in terms of conceptual (referring to the conceptual formulation of the evaluation), idiomatic (different linguistic expressions), semantic (differences related to the test content) and experiential (related to cultural differences) equivalence,^(25)^ giving rise to the pre-final version of the scale.

For the semantic and idiomatic analyses, the experts marked the item as unchanged if it was fully similar to the original scale item, as slightly changed if some words of the item were synonymous terms, and as heavily changed if there were words that would change the context of the item and if there were no synonymous terms.

The expert committee also analyzed whether the content of the items was pertinent to each domain, whether it was appropriate for the Brazilian culture, and whether or not they agreed with the item. The experts could present suggestions for changes to more appropriate terms or words. An analysis was performed to verify the index of agreement among the experts, according to the formula (C/C+D) x 100 proposed by Pasquali,^(26)^ where A is the total number of agreements and D is the total number of disagreements.

The last stage of the study was to evaluate the problems encountered during the use of the instrument and offer solutions to facilitate its understanding. For this purpose, the pre-final version was used in a pre-test with children who met the following eligibility criteria: age between 1 month and 13 years, discharged from a pediatric ICU within a minimum of 24 hours, and hospitalized in the pediatric ward of the IMIP. Children who were previously dependent on technology, readmitted to the pediatric ICU ≥ 24 hours after discharge, or with prior physical disability, genetic or neurological diseases or syndromes that limited functionality were excluded. After the pre-test, adjustments were made, obtaining the Brazilian version of the FSS in Portuguese spoken in Brazil.

The Kolmogorov-Smirnov test was used to assess data normality. Data are reported in absolute numbers and percentages, medians, minimums and maximums, and means ± standard deviations. Intraclass correlation coefficient (ICC) values were calculated to evaluate the reliability between the two evaluators. An ICC above 0.75 indicated good to excellent reliability.^(27)^

## RESULTS

When preparing the synthesized version of the translations, the authors considered a combination of the translations performed by the two translators. When the translations were different, the most common terms were used. For some items, changes were made in the phrases suggested by the translators to improve the semantic equivalence between the original scale items and the Brazilian version of the FSS.

The expert committee evaluated the semantic equivalence between the items of the original scale and the synthesized versions of the translations and back-translations (Table 1). It was found that eight items were considered slightly changed in the synthesized version of the translations and ten items were considered slightly changed in the synthesized version of the back-translations, compared to the original version.

**Table 1:** Evaluation of the semantic equivalence between the original and the synthesis of the versions translated into Portuguese and the synthesis of the back-translations of the Functional Status Scale

Expert opinion	STV Number of items (%)	SBTV Number of items (%)
Unchanged	33 (80.48)	31 (75.60)
Slightly changed	8 (19.51)	10 (24.39)
Heavily changed	0	0

STV - synthesis of the Portuguese translation versions; SBTV - synthesis of the back-translation versions.

In the content analysis (conceptual and of items), the agreement index was > 80% in most items. Only three items had an agreement index < 50% and were replaced, namely "suction", "continuous treatment with positive airway pressure" and the symbol and number "≥ 2". The modifications are shown in table 2.

**Table 2:** Description of items changed after evaluation of the translation of the Functional Status Scale by the expert committee

Original version	Synthesis of translations	Final version
Oxygen treatment and/or suctioning	*Tratamento com oxigênio e/ou sucção *	*Tratamento com oxigênio e/ou aspiração de vias aéreas*
Continuous positive airway pressure for all or part of the day and/or mechanical ventilatory support for part of the day	*Tratamento contínuo com pressão positiva nas vias aéreas durante todo ou parte do dia e/ou suporte ventilatório mecânico durante parte do dia *	*CPAP durante todo ou parte do dia e/ou suporte ventilatório mecânico durante parte do dia*
≥ 2 limbs functionally impaired	*≥ 2 membros com deficiência funcional *	*Dois ou mais membros com deficiência funcional*

CPAP - continuous positive airway pressure.

The Brazilian version of the FSS is presented in table 3 and was used in the pre-test stage. It was applied in a printed form, in an in-hospital observation scenario. In the administration of the FSS, the evaluated items were observational, but some of them were asked to the child or the caregivers because at the time of the evaluation, they could not be observed. For example, in the first item of the mental state domain, it was asked if the child had a normal sleep/wake pattern; in the feeding domain, how the child was eating was asked so that the evaluators could score it on the scale. For the application of the scale, approximately 5 to 10 minutes were necessary for each evaluation. No items were modified after the pre-test was performed, and the pre-final version thus constitutes the final version of the scale.

**Table 3:** Brazilian version of the pediatric Functional Status Scale

	Normal (Pontos = 1)	Disfunção leve (Pontos = 2)	Disfunção moderada (Pontos = 3)	Disfunção grave (Pontos = 4)	Disfunção muito grave (Pontos = 5)
Estado mental	Períodos normais de sono/vigília; responsividade adequada	Sonolento, mas suscetível ao ruído/toque/ movimento e/ou períodos de não responsividade social	Letárgico e/ou irritável	Despertar mínimo aos estímulos (estupor)	Coma não responsivo, e/ou estado vegetativo
Funcionalidade sensorial	Audição e visão intactas e responsivo ao toque	Suspeita de perda auditiva ou visual	Não reativo a estímulos auditivos ou a estímulos visuais	Não reativo a estímulos auditivos ou a estímulos visuais	Respostas anormais à dor ou ao toque
Comunicação	Vocalização apropriada, não chorando, expressividade facial ou gestos interativos	Diminuição da vocalização, expressão facial e/ou responsividade social	Ausência de comportamento de busca de atenção	Nenhuma demonstração de desconforto	Ausência de comunicação
Funcionamento motor	Movimentos corporais coordenados, controle muscular normal, e consciência da ação e da reação	1 membro com deficiência funcional	Dois ou mais membros com deficiência funcional	Controle deficiente da cabeça	Espasticidade difusa, paralisia ou postura de decerebração/decorticação
Alimentação	Todos os alimentos ingeridos por via oral com ajuda adequada para a idade	Nada por via oral ou necessidade de ajuda inadequada para a idade com a alimentação	Alimentação via oral e por tubo	Nutrição parenteral com administração por via oral ou por tubo	Nutrição parenteral exclusiva
Estado respiratório	Ar ambiente e sem suporte artificial ou dispositivos auxiliares	Tratamento com oxigênio e/ou aspiração de vias aéreas	Traqueostomia	CPAP durante todo ou parte do dia e/ou suporte ventilatório mecânico durante parte do dia	Suporte ventilatório mecânico durante todo o dia e toda a noite

CPAP - pressão positiva contínua nas vias aéreas.

The results of the pre-test stage refer to the evaluation of two researchers who applied the scale in 25 children. There was very good reliability among the observers for the FSS total score, with an ICC (95% confidence interval) of 0.85 (0.59 - 0.95). The demographic characteristics of the evaluated population are shown in table 4. The minimum score achieved by the FSS was 6 points, and the maximum score was 13 points, resulting in a mean score of 7.48 ± 2.08, which indicated a level of functionality classified between adequate and mild dysfunction.

**Table 4:** Characteristics of the population submitted to the pre-test with the pediatric Functional Status Scale, Brazilian version (n = 25)

Variable	
Age (months)	18 (1 - 156)
Male	14 (56)
Length of stay in the pediatric ICU	6.96 ± 7.03
Needed IMV in the pediatric ICU	15 (60)
Needed oxygen therapy	13 (52)
Needed a tracheostomy	0
Needed sedation in the pediatric ICU	6 (24)
Needed NMB in the pediatric ICU	2 (8)
Diagnosis for admission to the pediatric ICU	
Cardiac surgery	11 (44)
Respiratory (pneumonia and bronchiolitis)	7 (2)
Sepsis (ARDS and diarrhea)	2 (8)
Abdominal surgery	2 (8)
Diarrhea	1 (4)
Leptospirosis	1 (4)
Ketoacidosis	1 (4)
FSS score	7.48 ± 2.08

ICU - intensive care unit; IMV - invasive mechanical ventilation; NMB - neuromuscular blocker; ARDS - acute respiratory distress syndrome; FSS - Functional Status Scale. Values expressed as median (minimum value and maximum value), number (%), or mean ± standard deviation.

## DISCUSSION

This study described the process of the translation and cross-cultural adaptation of the pediatric FSS into Portuguese spoken in Brazil, which resulted in the Brazilian version of this instrument. This is the first study to carry out the official translation and adaptation of the FSS. Although there is no gold standard template to follow for this process, four steps are essential and are reported in guidelines and recommendations: translation, back-translation, review by an expert committee and pre-testing.^(22,28)^ All steps were rigorously followed in this study to preserve social, cultural and linguistic characteristics and use regional terms.^(23,29)^

After the translation, back-translation, expert committee evaluation and pre-test stages, it was found that the translation and cross-cultural adaptation process was successful. The semantic, idiomatic, conceptual and cultural equivalences obtained between the original scale and the Brazilian Portuguese version were satisfactory, and few modifications were made for the items to be appropriate to the medical-hospital culture of Brazil. It is important to carry out this process so that the terms used in the instrument are consistent with the reality experienced by the target population and to attempt to preserve the psychometric properties of the original instrument.^(22,24,25)^

Silva et al.^(30)^ cross-culturally adapted into Portuguese the Functional Status Score for the ICU (FSS-ICU), which is an instrument for adults in intensive care; similar to the present study, the authors noted the relevance of the participation of different and bilingual translators to reduce the possibility of bias for the domains of the items studied. Different from the study by Silva et al., our study included the participation of an expert committee that used agreement indexes to adapt items that did not match the Brazilian culture. This index was used to provide numerical evidence of the agreement of the experts rather than relying solely on the subjective evidence of the expert's speech.

Analogous to the study by Silva et al.,^(30)^ during the pre-test, the evaluators did not report problems with doubts or interpretation difficulties affecting their performance, and because of this, no adjustments were made to the Brazilian version after the pre-test stage.

In this study, the interobserver reliability was tested for the total FSS score, and values very close to those from the original scale validation^(17)^ were observed. Interobserver reliability is a fundamental property because the FSS is an observational instrument; that is, the smaller the variation produced in repeated measurements, the greater its reliability is.^(27)^

The FSS for children is an instrument that can be used for physical evaluation in the pediatric ICU environment as well as in wards. It does not depend on subjective evaluations and, like the FSS-ICU,^(30)^ does not require any additional equipment. The pediatric FSS can also be easily integrated into the usual clinical care of the physical therapist, in addition to being an instrument with ease of understanding and clinical applicability.

## CONCLUSIONS

The Brazilian version of the Functional Status Scale was translated and cross-culturally adapted. It is a promising and useful tool for clinicians and researchers to evaluate the functional outcome of hospitalized children, mainly after discharge from a pediatric intensive care unit. Additional studies should be performed to evaluate the reproducibility and validity of the Functional Status Scale for the assessment of the psychometric properties of this instrument, in order to make it feasible for use in the different regions of Brazil.
